# Antiaversive Effects of Cannabinoids: Is the Periaqueductal Gray Involved?

**DOI:** 10.1155/2009/625469

**Published:** 2008-12-02

**Authors:** F. A. Moreira, D. C. Aguiar, A. C. Campos, S. F. Lisboa, A. L. Terzian, L. B. Resstel, F. S. Guimarães

**Affiliations:** Department of Pharmacology, School of Medicine of Ribeirão Preto, University of São Paulo, Avenida Bandeirantes 3900, 14049900 Ribeirão Preto, SP, Brazil

## Abstract

Cannabinoids play an important role in activity-dependent changes in synaptic activity and can interfere in several brain functions, including responses to aversive stimuli. The regions responsible for their effects, however, are still unclear. Cannabinoid type 1 (CB1) receptors are widely distributed in the central nervous system and are present in the periaqueductal gray (PAG), a midbrain structure closely involved in responses related to aversive states. Accordingly, exposure to stressful stimuli increases endocannabinoid (eCB) levels in the PAG, and local administration of CB1 agonists or drugs that facilitate eCB-mediated neurotransmission produces antinociceptive and antiaversive effects. To investigate if these drugs would also interfere in animal models that are sensitive to anxiolytic drugs, we verified the responses to intra-PAG injection of CB1 agonists in rats submitted to the elevated plus-maze, the Vogel punished licking test, or contextual aversive conditioning model. The drugs induced anxiolytic-like effects in all tests. The same was observed with the transient receptor potential vanilloid type 1 (TRPV1) antagonist capsazepine and with cannabidiol, a nonpsychotomimetic phytocannabinoid that produces anxiolytic-like effects after systemic administration in humans and laboratory animals. These results, therefore, suggest that the PAG could be an important site for the antiaversive effects of cannabinoids.

## 1. Introduction

*Cannabis sativa* plant has been used for various purposes since the dawn of civilizations [1, 2], although only in the middle of twentieth century were its chemical constituents identified. Among its major components, there are the phytocannabinoids cannabinol, cannabidiol (CBD), and Δ^9^-tetrahydrocannabinol (Δ^9^-THC), the latter accounting for most of cannabis effects [3–5]. The mechanisms of Δ^9^-THC effects started to be unveiled in the late 80s, with the discovery of CB1 receptors [6, 7]. Soon afterwards, the first endogenous agonist (arachidonoyl ethanolamide, AEA) was isolated and named anandamide, after the Sanskrit word “ananda” for “bliss” [8]. A second endocannabinoid, 2-arachidonoyl glycerol [9], and another cannabinoid receptor, called CB2 [10], soon followed. Selective antagonists were developed, such as rimonabant and AM251, supporting the notion that the CB1 receptor is the major responsible for the behavioral effects of cannabinoids [11, 12]. The expression of this receptor is considerably high in several brain regions such as the basal ganglia, cerebral cortex, hippocampus, amygdale, hypothalamus, and periaqueductal gray (PAG) [13, 14].

CB1 receptors are believed to be located in presynaptic terminals [15]. They activate Gi proteins that inhibit adenylate cyclase and calcium channels and enhance potassium currents, thereby reducing neural firing and neurotransmitter release [16]. This complements the fact that endocannabinoids are synthesized on a stimulus-dependent manner in postsynaptic neurons and immediately diffuse to the synaptic cleft [16]. Thus, contrary to classical neurotransmitters, endocannabinoids act “on demand” as retrograde messengers, inhibiting neural activity. Their effects cease by internalization followed by hydrolysis in neurons. It is still controversial whether endocannabinoids move through the cell membrane passively or are internalized by a putative transporter. Although the latter remains to be identified [17, 18], pharmacological tools were developed, such as AM404, which are able to inhibit it and, thereby, increase CB1 receptor activation by AEA [18]. Inside neurons, AEA and 2-AG are catabolized by fatty acid amide hydrolase (FAAH) and monoacyl glycerol lipase (MGL), respectively [19]. Possibly, FAAH is located in postsynaptic neurons, whereas MGL is expressed in the presynapse [17]. Selective inhibitors of either FAAH (URB597) or MGL (URB602) have been developed, which provide the possibility of enhancing CB1 receptor activation by increasing the brain levels of endocannabinoids. Studies with these drugs as well as with genetically modified mice have related endocannabinoids to several functions of the central nervous system (for review, see [20]).

Other putative components of this system are the transient receptor potential vanilloid type 1 (TRPV1), the peroxisome-proliferator activated receptor, and the G protein-coupled receptor GPR55. Although anandamide binds to all these receptors, their functions remain uncertain [21]. In addition, an allosteric site in the CB1 receptor has been identified [22] and there is the possibility that, contrary to the initial thoughts, CB2 receptors may indeed be relevant for behavioral responses [23, 24]. Finally, more substances have been proposed as endocannabinoids, such as arachidonoyl dopamine, virodhamine, and noladin ether [20].

## 2. Cannabinoids and Anxiety

Natural or synthetic cannabinoids or CB1 receptor antagonists often yield complex responses in experimental models of anxiety. As summarized in Table 1, several authors have noticed bell-shaped dose-response curves in animal models predictive of anxiogenic- or anxiolytic-like activity, namely, the elevated plus maze (EPM), the elevated zero maze (EZM), the light dark test (DLT), and the Vogel conflict test (VCT). CB1 receptor agonists tend to be anxiolytic in lower doses, whereas higher doses may be anxiogenic [25]. However, compounds that enhance endocannabinoid effects, such as inhibitors of AEA uptake or hydrolysis, appear to produce only anxiolytic effects without bell-shaped dose-response curves (Table 1).

The reasons for these complex effects remain unknown. One possibility could be that these drugs would interfere with diverse brain regions which have different roles in the modulation of anxiety-like responses. However, the sites responsible for the effects of cannabinoids remain poorly investigated. CB1 receptors, as well as the putative protein responsible for internalization of AEA and the enzyme FAAH, are expressed in several regions of CNS related to anxiety, aversion, and defensive behaviors, including the prefrontal cortex, amygdala, hippocampus, hypothalamus, and PAG [13, 14]. These structures are proposed to be part of a system responsible for the elaboration of behavioral and autonomic responses to aversive stimuli. They are possible neural sites whose malfunction would lead to psychiatric pathologies such as generalized anxiety and panic disorders [26]. In this context, anxiolytic drugs would act by normalizing the functions of these structures [27, 28]. Moreover, this brain aversive system would be responsible for behavioral suppression in animal models predictive of anxiolytic-like activity. Generally, models of experimental anxiety rely on exposing animals to situation that generates conflicts between approach and avoidance, which can be generated by the drive of exploring a new, though, aversive environment, or by a source of reward that is associated with punishment. Anxiolytic-like drugs injected either systemically or into these structures shift the conflict toward approach responses [27, 28]. Thus, these models provide invaluable insights into the neurobiology of anxiety and the pharmacology of anxiolytic compounds. As discussed below, we have used direct drug administration in animals submitted to these models for studying the possible role of the PAG in the antiaversive actions of cannabinoids.

## 3. Anxiolytic Effects of Cannabinoids in the Periaqueductal Gray

The PAG is a mesencephalic structure that surrounds the cerebral aqueduct and can be divided along its rostrocaudal axis into dorsomedial, dorsolateral (dlPAG), lateral, and ventrolateral columns [29]. It is an important site in ascending pain transmission and a major component of a descending pain inhibitory system. Moreover, this structure receives glutamatergic projection from forebrain regions and sends descendent pathways to motor outputs and to autonomic centres that control blood pressure and heart rate [26]. The dorsal columns (dPAG) are possibly responsible for the elaboration of active defensive behaviors (see [26], for review). Lesions of the dPAG inhibit fear and anxiety produced by stimulation of the amygdala whereas stimulation of this region induces threat display associated with vocalization and strong flight responses [26]. In the caudal ventrolateral PAG, however, immobility has been described as the main outcome of local stimulation [30].

CB1 receptors are distributed along the various columns of this structure [13]. Moreover, administration of CB1 agonists increases Fos expression [31] and brain metabolic activity in the PAG of rats [32], suggesting that this structure could be involved in the effects of systemically administered cannabinoids. In agreement with this proposal, injection of CB1 receptor agonists into the dlPAG of rats has been shown to induce antinociceptive responses [33] and electric stimulation of the dorsal and lateral columns induces antinociception via activation of CB1 receptors accompanied by local AEA release [34]. Furthermore, subcutaneous formalin injection, a painful stimulus, substantially increased the release of AEA in the PAG [34, 35].

Concerning the possible involvement of PAG-endocannabinoid system on modulation of anxiety-like behaviors, an initial study showed that local administration of HU210, a potent CB1 agonist, attenuated the flight responses induced by dPAG injections of the excitatory amino acid D,L-homocysteic acid (see [36, Table 2]). In a subsequent study, where the injections were restricted to the dorsomedial PAG, HU210 decreased hyperlocomotion induced by aversive ultrasound stimulation, but failed to change freezing responses. Moreover, HU210 effects were not entirely blocked by previous local injection of a CB1 receptor antagonist [70].

Considering these initial results, we decided to further investigate a possible influence of the PAG-endocannabinoid system on anxiety-like behaviors in rats submitted to different animal models of anxiety (Table 2). First, we showed that AEA injected into the dlPAG increased the exploration of the open arms of the elevated plus maze (EPM) [71], a model based on a natural conflict between exploratory behavior and innate fear of open spaces. The effects of AEA were similar to those observed with classical anxiolytic benzodiazepines [72] and were blocked by previous treatment with AM251, a CB1 receptor antagonist. These effects were also potentiated by previous treatment with AM404, an inhibitor of AEA uptake/metabolism. AM404 by itself, however, was without effect in this model. AEA produced an inverted U-shaped dose-response curve, with higher doses being ineffective [71].

To confirm a possible anticonflict effect of AEA in the dlPAG, we used the Vogel conflict test (VCT) [73], an animal model of anxiety not based on innate fear but instead on suppression of punished responses learned during the test. In this model, water-deprived rodents are exposed to a conflict between licking the spout of a bottle containing water and receiving a mild shock on the tong [74]. Anxiolytics that potentiate the action of *γ*-aminobutyric acid such as the benzodiazepines typically increase the number of punished licks [75]. AEA also induced anxiolytic-like effects in the VCT at the same dose range observed in the EPM (Table 2). Different from the results obtained in the latter model, AM404 was also able to increase the number of punished licks (Table 2). Although the causes of these contradictory results are not clear, they could involve the distinct animal models of anxiety employed. Brain endocannabinoids have been proposed to act as a “stress buffer system” [76], recruited by highly demanding situations. It was possible that the VCT, by involving pain and water deprivation, engages the endocannabinoid system in the dlPAG to a greater extent than the EPM. Actually, as discussed above, painful stimuli such as those used in the VCT have already been showed to increase AEA in this region [77].

We have further investigated this effect by intra-dlPAG administration of AEA and AM404 in rats submitted to a contextual fear conditioning paradigm, an animal model that also involves pain exposure [78]. Animals re-exposed to an environment where they had being previously submitted to an aversive stimulation, such as electrical footshocks, show behavioral and cardiovascular changes characterized by immobility (freezing) and mean arterial pressure (MAP) and heart rate (HR) increases [79, 80]. Although electrical or chemical stimulation of the dorsal portion of PAG is usually related with flight reactions, it can also produce freezing responses and increased cardiovascular activity [26]. Re-exposure to an aversively conditioned context increases neuronal activity in the PAG [81, 82], and PAG lesions block freezing to aversively conditioned stimulus [83, 84]. dlPAG microinjection of AEA or AM404 blocked the expression of the conditioned aversive responses [78]. This effect was inhibited by local pretreatment with AM251, reinforcing the involvement of CB1 receptors.

Altogether, these results suggest that the endocannabinoid system in the dlPAG can modulate responses to aversive stimuli. The mechanisms of these effects are still unclear. Using brain slices of the rat PAG, Vaughan et al. [85] showed that cannabinoids act via CB1 receptors to inhibit GABAergic and glutamatergic synaptic transmission. The efficacy of endogenous cannabinoids was limited by uptake and breakdown since AEA was only able to inhibit evoked inhibitory postsynaptic currents in the presence of the AT inhibitor, AM404. Several studies indicate that GABA- and glutamate-mediated neurotransmissions in the dPAG play opposite roles. While the former tonically inhibits defensive responses, the latter facilitates them [26]. Thus, CB1-mediated inhibitory effects on these two neurotransmitter systems could be one of the explanations for the observed bell-shaped dose-response curve induced by AEA in this region as well as the contradictory results regarding the effects of cannabinoids on anxiety (see Table 1 and text bellow for a discussion on the possible involvement of TRPV1 receptors).

These mechanisms may explain the effects in the PAG, yet they do not necessarily apply to other brain regions. In some areas, the levels of CB1 receptor expression can be higher in GABAergic (particularly in cholecystokinin-containing basket cells) as compared to glutamatergic neurons, with cannabinoid effects favoring impairment of inhibitory mechanisms mediated by the former neuronal population [16]. However, it remains to be further investigated how these neural subpopulations contribute to specific behavioral effects of cannabinoids. In addition, GABAergic and glutamatergic neurons may have different sensitivity to CB1 agonists or antagonists depending on the species under investigation. For instance, Haller et al. [52] observed opposite effects in mice and rats tested with the same doses of a cannabinoid in models of anxiety-like behavior (see Table 1). Inhibitory and excitatory currents were differentially affected in the hippocampi of these species, providing a possible basis for the discrepancies in the behavioral responses. Since we have employed rats as subjects in all our experiments, studies in other species could further consolidate our hypothesis that glutamatergic and GABAergic inhibitions would mediate anxiolytic- and anxiogenic-like effects of cannabinoids, respectively. For a more extensive discussion on the relevance of diverse neural subpopulations for the effects of cannabinoids, see [89].

## 4. Cannabidiol

Cannabidiol (CBD) is a major nonpsychotomimetic constituent of *Cannabis sativa* that is able to antagonize the anxiogenic and psychotomimetic effects of high doses of Δ^9^-THC [90, 91]. It also promotes anxiolytic-like effects in several animal models (see [44–47], Table 1). In addition, CBD induces anxiolytic effects in healthy volunteers in the simulated public speaking test, a model of clinical anxiety, and in subjects submitted to a functional imaging analysis study [92, 93]. However, as commonly seem with other cannabinoids in animal models of anxiety, experiments with CBD yield bell-shaped dose-response curves, low doses being anxiolytic, and higher doses being ineffective [44]. The mechanisms for these actions remain poorly understood. CBD has low affinity for CB1 or CB2 receptors and could facilitate the endocannabinoid signalling by inhibition of AEA uptake or its enzymatic hydrolysis. It can also act as an agonist of TRPV1 or 5HT1A receptors [94, 95].

Considering that the PAG, in addition to CB1 [14], also expresses a significant number of TRPV1 and 5HT1A receptors [96, 97], we decided to verify if this region could be related to the effects of CBD. We found that CBD microinjections into the dlPAG produced anxiolytic-like effects in rats submitted to the EPM or the VCT [86] (Table 2). The effects in the EPM, however, also showed a bell-shaped dose-response curve, but were not blocked by previous local administration of AM251 [86], employed at the same dose that was able to antagonize the anxiolytic-like effects of AEA and AM404 (Table 2). The anxiolytic-like effects of CBD, however, were prevented by WAY100635, an antagonist of 5HT1A receptors. Activation of these Gi-coupled-receptors enhances K+ currents and inhibits adenylyl cyclase activity [98]. They act as inhibitory autoreceptors in serotonergic neurons in the raphe nuclei but are also localized postsynaptically in several brain regions, including the PAG, amygdala, hippocampus, and frontal cortex. Actually, the PAG receives serotonergic projections from the dorsal raphe nuclei, and local activation of 5HT1A receptors promotes the control of anxiety states and the hypothalamus-pituitary-adrenal axis during stress responses [99]. Thus, 5HT1A receptors located in the PAG are possibly involved in the anxiolytic-like effects of CBD, a hypothesis corroborated by several studies showing that agonists of these receptors produce anxiolytic effects in the PAG [100, 101].

## 5. TRPV1 Receptors Methods

TRPV1 receptors belong to a large family of calcium-permeable cation channels [102]. They can be activated by elevation in temperature, pH decrease, or by exogenous ligands such as capsaicin, the pungent ingredient of red hot chilli peppers [103, 104]. They have been related to pain transmission and inflammatory responses in the peripheral nervous system. In addition to environmental stimuli, endocannabinoids such as AEA and N-arachidonyldopamine can also activate TRPV1 receptors. As a consequence, they can also be denominated endovanilloids [104, 105].

TRPV1 receptors are expressed in various brain regions related to anxiety, including the PAG [106, 107], where they can regulate glutamate release. Corroborating this proposal, local infusion of capsaicin produces antinociception by increasing glutamate release in this region [108]. In addition, activation of presynaptic TRPV1 receptors produced an excitatory effect in the firing activity of dlPAG neurons [109]. Glutamate is the main excitatory neurotransmitter in the central nervous system, and the injection of NMDA antagonist receptors into the dlPAG promotes anxiolytic effects in the EPM and VCT [110].

Few studies, however, have investigated the role of TRPV1 in anxiety. Systemic administration of capsazepine, a TRPV1 antagonist, induced anxiolytic-like effects in rats submitted to the EPM (Table 1) [69]. More recently, Marsch et al. [111] demonstrated that TRPV1-deficient mice show decreased anxiety in the EPM and light-dark test. Accordingly, the dual FAAH/TRPV1 blocker N-arachidonoyl-serotonin is able to induce CB1-mediated anxiolytic-like effects more potently than selective blockers of FAAH or TRPV1, further suggesting opposite roles for CB1 and TRPV1 receptors [112].

To further investigate the role of TRPV1 on anxiety modulation, we verified the effects of intra-dlPAG injection of capsazepine in rats submitted to the EPM and VCT. This drug decreased anxiety-like behaviors in both models (Table 2), suggesting that TRPV1 receptors facilitate anxiety responses in the PAG. The fact that AEA and CBD can also activate TRPV1 receptors [94, 104, 105] could help to explain the bell-shaped dose-response curves usually found with these compounds regarding their anxiolytic effects (Tables 1 and 2). In agreement with this proposal, it was recently showed that capsazepine blocks the anxiogenic effects of high doses of AEA in the prefrontal cortex [113]. It remained to be tested if similar effects could occur in the dlPAG. In an initial study, we confirmed this possibility, showing that intra-dlPAG pretreatment with an ineffective dose of capsazepine was able to turn the higher, ineffective dose of the CBD into an anxiolytic one (Table 2).

## 6. Conclusions

The pieces of evidence revised above suggest that the PAG, particularly its dorsolateral column, is involved in the modulatory effects of cannabinoids on defensive responses. This does not mean that the PAG is the only or the most relevant structure accounting for the antiaversive properties of cannabinoids. Other authors have also identified brain sites where CB1 receptor activation induces anxiolytic-like effects. Injection of low doses of Δ^9^-THC either in the ventral hippocampus (5 *μ*g) or in the prefrontal cortex (10 *μ*g) resulted in anxiolytic-like effects; whereas in the amygdala (1 *μ*g), opposite results were reported [114]. An early work has also shown anxiogenic-like effect of Δ^9^-THC in this brain region [115] Moreover, intraprefrontal cortex injection of low or high doses of methanandamide induces CB1-mediated anxiolytic- or TRPV1-mediated anxiogenic-like effects, respectively [113]. Other authors have also investigated brain sites mediating nociceptive responses, antidepressive-like activity, and rewarding effects of cannabinoids [89].

**Figure 1:**
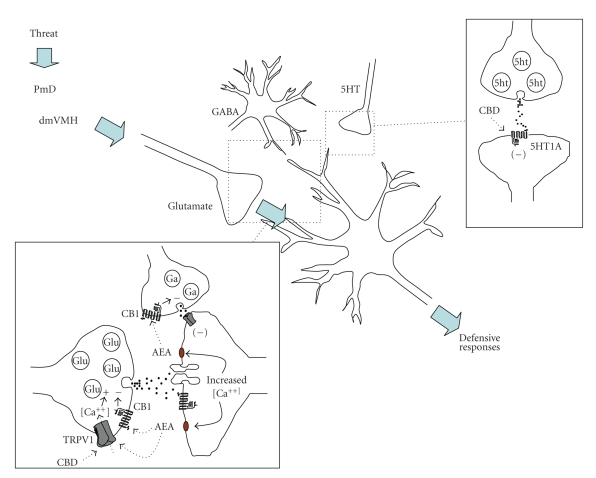
Possible effects of cannabinoids in the dlPAG. Glutamatergic inputs from forebrain structures such as the dorsomedial part of the ventromedial hypothalamic nucleus (dmVMH) and dorsal premammilary hypothalamic nucleus (PmD) activate a local neural substrate that mediates defensive responses [88]. This substrate is under GABAergic and serotonergic inhibitory influence [26]. Activation of CB1 receptors by cannabinoids such as AEA interferes with presynaptic glutamate (Glu) and GABA (Ga) neurotransmitter release. CB1-mediated decrease of glutamate release would promote anxiolytic-like effects. Activation of TRPV1 presynaptic receptors, on the other hand, would produce opposite effects. The anxiolytic effects of cannabidiol (CBD), a nonpsychotomimetic cannabinoid, in the dlPAG are not mediated by CB1 receptors, but probably involve activation of postsynaptic 5HT1A receptors. The bell-shaped dose-response curves observed with AEA and CBD may depend on activation of TRPV1 receptors. Regarding AEA, a presynaptic decrease of GABA release could also be related to this effect.

In conclusion, local administration of CB1 agonists into the dlPAG produces anxiolytic-like effects in several animal models. These effects are prevented by AM251, indicating that they are being mediated by activation of CB1 receptors, possibly by presynaptic inhibition of glutamate release (see Figure 1). Results with AM404, an AEA metabolism/uptake inhibitor, also suggest that local synthesis of endocannabinoids in the dlPAG can modulate defensive responses, at least under high-aversive conditions. The results also showed that the dlPAG could be involved in the reported anxiolytic effects of CBD, a nonpsychotomimetic phytocannabinoid. This compound, however, appears to act by activating 5HT1A receptors (Figure 1). Finally, activation of vanilloid TRPV1 receptors in the dlPAG seems to facilitate defensive responses (Figure 1) and may be, in part, responsible for the bell-shaped dose-response curves of the anxiolytic effects of AEA and CBD. A balance between CB1- and TRPV1-activations is a possible mechanism through which endogenous AEA could control aversive responses.

## Figures and Tables

**Table 1:** Effects of cannabinoids and drugs that interfere with the endocannabinoid system in animal models predictive of anxiolytic- or anxiogenic-like activity. (AEA: AEA; Δ^9^-THC: Δ^9^-tetrahydrocannabinol; CBD: cannabidiol; EPM: elevated plus-maze; EXM: elevated X-maze; EZM: elevated zero-maze; VCT: Vogel conflict test; FC: fear conditioning; DLT: dark-light test; SI: social; NSF: novelty-suppressed feeding interaction.)

Drug	Test	Dose (species)		Reference
		Anxiolytic-like effects	Anxiogenic-like effects	
Phytocannabinoids				

Δ^9^-THC	EPM		10–20 mg/kg (mouse)	[37]
	EPM		1–10 mg/kg (rat)	[37]
	DLT	0.3 mg/kg (mouse)	0.5 mg/kg (mouse)	[38]
	DLT	0.3 mg/kg (mouse)		[39]
	EPM		1–10 mg/kg (mouse)	[40]
	EPM	0.075–0.75 mg/kg (rat)		[41]
	EPM		0.5–2.5 mg/kg (rat)	[42]
	DLT		1.25–2.5 mg/kg (rat)	[42]
	EPM	0.075–1.5; 3* mg/kg (rat)		[43]

CBD	EPM	1–10 mg/kg (mouse)		[37]
	EPM	2.5–10; 20* mg/kg (rat)		[44]
	EPM	5 mg/kg (rat)		[45]
	VCT	10 mg/kg (rat)		[46]
	FC	10 mg/kg (rat)		[47]

CB1 agonists				

HU210	EXM		25 *μ*g/kg, 10 days (rat)	[48]
	NSF	100 *μ*g/kg/day–10 days (rat)		[49]
	EPM	10 *μ*g/kg (rat)	50 *μ*g/kg (rat)	[50]

WIN-55212	EPM	1–3 mg/kg (mouse)		[51]
	EPM	1–3; 10* mg/kg (mouse)		[40]
	EPM	1–3 mg/kg (mouse)	1–3 mg/kg (rat)	[52]

CP55940	EPM		75–125 *μ*g/kg (rat)	[53]
	EPM		75 *μ*g/kg (rat)	[54]
	EPM	1 *μ*g/kg (rat)	50 *μ*g/kg (rat)	[55]
	EPM	2.5–5 *μ*g/kg (rat)	40 *μ*g/kg (rat)	[56]
	SI		40 *μ*g/kg (rat)	[57]
	EPM	0.1–0.3 mg/kg (mouse)		[40]

AEA	EPM		10 mg/kg (mouse)	[58]
	DLT	0.3 mg/kg (rat)		[59]

AEA uptake inhibitor				

AM404	EPM	5 mg/kg (rat)		[60]
	EPM	1–3; 10* mg/kg (mouse)		[40]
	EPM	0.75–1.25 mg/kg (rat)		[37]

AEA metabolism inhibitors				

URB597	EZM	0.1 mg/kg (rat)		[61]
	EPM	0.1–0.3 mg/kg (mouse)		[40]
	EPM	0.1 mg/kg (mouse)		[62]
	DLT	0.1–0.3 mg/kg (rat)		[55]
	EPM	1 mg/kg (mouse)		[63]

AACOCF_3_	DLT	4 mg/kg (mouse)		[64]

CB1 antagonists				

Rimonabant	EPM		3 mg/kg (rat)	[65]
	EPM		3 mg/kg (rat)	[53]
	EPM	3 mg/kg (mouse)		[66]
	VCT	0.3–3 mg/kg (rat)		[67]
	EPM	10 mg/kg (rat)		[67]
	EPM		3–10 mg/kg (mouse)	[40]

AM251	EPM		3 mg/kg (mouse)	[51]
	EPM		1.3–3 mg/kg (mouse)	[68]
	EPM		3–10 mg/kg (mouse)	[40]
	EPM		1–3 mg/kg (mouse)	[52]

TRPV1 agonists				

Olvanil	EPM	5 mg/kg (rat)		[69]

TRPV1 antagonists				

Capzasepine	EPM	1–10 *μ*g/kg (rat)		[69]

*Bell-shaped dose-response curve.

**Table 2:** Effects of Cannabinoid-related drugs injected into the PAG of rats submitted to animal models of anxiety-related behaviors. (AEA: anandamide; ACEA: arachidonyl-2-chloro-ethylamide; CBD: cannabidiol; EPM: elevated plus-maze; VCT: Vogel conflict test; CFC: contextual fear conditioning; dlPAG: dorsolateral PAG; dPAG: dorsal (dorsolateral + dorsomedial) PAG; dmPAG: dorsomedial PAG; unpub: unpublished data.)

	Drug	PAG column	Test	Doses tested	Effect (effective dose)	Ref.
Phytocannabinoids	CBD	dlPAG	EPM, VCT	15–60 nmol	Anxiolytic (30 nmol*)	[86]

Endocannabinoids	AEA	dlPAG	EPM	0.05–50 pmol	Anxiolytic (5 pmol*^,1^)	[71]
			VCT	5 pmol	Anxiolytic	[73]
			CFC	5 pmol	Anxiolytic	[78]

CB1 receptor agonists	ACEA	dlPAG	EPM	0.05–5 pmol	Anxiolytic (0.05 pmol*)	[71]
	HU210	dPAG	dPAG chemical stimulation	0.1–5 *μ*g	Attenuated flight responses (0.1–5 *μ*g)	[36]
	HU210	dmPAG	Ultrasound-induced hyperlocomotion and freezing	5 *μ*g	Decreased hyperlocomotion, but increased freezing**	[70]

CB1 receptor antagonist	AM251	dlPAG	EPM, VCT, CFC	1–300 pmol	No effect by itself, but blocked AEA and AM404 anxiolytic effects	[71, 73, 78]
	Rimonabant	dPAG	Ultrasom-induced hyperlocomotion and freezing	30 *μ*g	No effect by itself	[70]

AEA uptake inhibitor	AM404	dlPAG	EPM	0.5–50 pmol	No effect by itself; potentiated the anxiolytic effect of AEA	[71]
			VCT	50 pmol	Anxiolytic	[73]
			CFC	50 pmol	Anxiolytic	[78]

AEA metabolism inhibitor	URB597	dlPAG	VCT	0.01–0.1 nmol	Anxiolytic (0.01 pmol*)	[73]

TRPV1 antagonists	Capsazepine	dlPAG	EPM, VCT	10–60 nmol	Anxiolytic (60 nmol)^+^	[87]

*Bell-shaped dose-response curve. Anxiolytic effect blocked by AM251 (100 pmol) and potentiated by AM404 (50 pmol). ^+^Capsazepine 10 nmol turned the higher, ineffective dose of CBD (60 nmol) into an anxiolytic one in the EPM [85]. **Not blocked by rimonabant 30 *μ*g.
